# Methylation of *DIRAS1* promotes colorectal cancer progression and may serve as a marker for poor prognosis

**DOI:** 10.1186/s13148-017-0348-0

**Published:** 2017-05-10

**Authors:** Ruipan Zheng, Dan Gao, Tao He, Meiying Zhang, Xiaomei Zhang, Enqiang Linghu, Lixin Wei, Mingzhou Guo

**Affiliations:** 1 Department of Gastroenterology and Hepatology, Chinese People’s Liberation Army General Hospital, #28 Fuxing Road, Beijing, 100853 China; 2 Department of Pathology, Chinese People’s Liberation Army General Hospital, 28 Fu-Xing Road, Beijing, 100853 China; 3 School of Medicine, Nankai University, #94 Weijin Road, Tianjin, 300071 China

**Keywords:** *DIRAS1*, Epigenetics, DNA methylation, Colorectal cancer

## Abstract

**Background:**

*DIRAS1* is a new member of the Ras gene family. It was described as a potential tumor suppressor in human glioblastomas and esophageal cancer. The role of *DIRAS1* in colorectal cancer remains unclear.

**Methods:**

To explore the epigenetic changes and function of *DIRAS1* in human colorectal cancer, we studied ten colorectal cancer cell lines and 146 primary colorectal cancer samples and 50 matched adjacent samples using semi-quantitative reverse transcription PCR, immunohistochemistry, methylation-specific PCR and bisulfite sequencing, western blot, flow cytometry, and transwell assays.

**Results:**

*DIRAS1* expression was found in DKO and HCT116 cells, while reduced expression was detected in LoVo, SW48, LS180, and SW620 cells, and there was no expression detected in DLD1, HT29, RKO, and SW480 cells. Complete methylation was found in the promoter region of DLD1, HT29, RKO, and SW480 cells. Partial methylation was detected in LoVo, LS180, SW48, and SW620 cells, and unmethylation was found in DKO and HCT116 cells. These results indicate that promoter region methylation correlated with loss of/reduced expression of *DIRAS1*. Re-expression of *DIRAS1* was induced by 5-aza-2′-deoxycytidine, suggesting that the expression of *DIRAS1* is regulated by promoter region methylation. *DIRAS1* was methylated in 47.3% (69/146) of primary colorectal cancer samples, no methylation was found in non-cancerous colonic tissue samples. Methylation of *DIRAS1* was significantly associated with TNM stage (*P <* 0.05) and short survival time (*P =* 0.0121). *DIRAS1* induced apoptosis and inhibited cell proliferation, migration, and invasion in colorectal cancer. Finally, *DIRAS1* suppressed colorectal cancer cell xenograft growth in nude mice.

**Conclusions:**

*DIRAS1* is frequently methylated in human colorectal cancer and the expression of *DIRAS1* is regulated by promoter region methylation. Methylation of *DIRAS1* is a marker of poor prognosis in human colorectal cancer.

**Electronic supplementary material:**

The online version of this article (doi:10.1186/s13148-017-0348-0) contains supplementary material, which is available to authorized users.

## Background

Colorectal cancer (CRC) is the third most common malignancy and the fourth leading cause of cancer-related death worldwide [1–3]. Genetic alterations are common in CRC cells, while they are rare in normal cells [4]. Hereditary forms of colorectal cancer account for less than 5% of colorectal cancer [5]. Inactivation of gene function by germline mutations within at least one of four mismatch repair genes (MLH1, MSH2, MSH6, and PMS2) can be found in approximately 70% of cases, and 95% of the mutations occur in hMSH2 or hMLH1 [6]. In sporadic cancer, DNA damage repair genes are rarely found to be mutated. Researchers have confirmed that promoter region methylation accounts for 80–90% of MLH1 inactivation in sporadic MSI-H (high-level microsatellite instability) colorectal cancer [7]. Thus, DNA methylation is increasingly recognized as a common mode of gene inactivation in DNA damage repair and other signaling pathways. Different genes were found to be frequently methylated in human colorectal cancer [8–11]. Some studies demonstrated that somatic epigenetic dysregulation occurs not only in cancer tissues but also in noncancerous and preneoplastic tissues. These studies suggest that epigenetic events are potentially more promising somatic CRC risk markers than gene mutations. Age and environmental factors are regarded as impact factors of epigenetics [4].

*DIRAS1* is a distinct subfamily member of Ras GTPases. *DIRAS1* is also known as *Rig* (Ras-related inhibitor of cell growth), which is located in chromosome 19p13.3 [12]. *DIRAS1* has been described as a potential tumor suppressor in human glioblastomas and esophageal cancer, and downregulation of the *DIRAS1* predicts poor prognosis in esophageal squamous cell carcinoma [12, 13]; however, its role in colorectal cancer remains unclear. Therefore, we analyzed the epigenetic regulation and function of *DIRAS1* in human colorectal cancer.

## Methods

### Cell lines and tumor specimens

Human colorectal cancer lines, DKO (DNMT1 and DNMT3b double knockout from HCT116 cells, a generous gift from Stephen Baylin), DLD1, HCT116, HT29, LoVo, LS180, RKO, SW48, SW480, and SW620 cells were previously established from primary colorectal cancer tissue samples and cultured as described previously [10].

Primary colorectal cancer samples (146) and matched adjacent samples (50) were collected during surgical resection at the Chinese PLA General Hospital. Twenty-six cases of formalin-fixed paraffin-embedded tumor tissues were available with matched adjacent tissue samples. All samples were collected under the guidelines approved by the institutional review board of the Chinese PLA General Hospital.

### 5-Aza-2′-deoxycytidine treatment

Colorectal cancer cell lines were split to a low density (30% confluence) 12 h before treatment. Cells were treated with 5-aza-2′-deoxycytidine (DAC, Sigma, St. Louis, MO) at a concentration of 2 μM. Growth medium conditioned with DAC at a concentration of 2 μM was exchanged every 24 h for a total of 96 h of treatment.

### RNA isolation and semi-quantitative reverse transcription PCR

Total RNA was isolated by Trizol reagent (Life Technologies, MD, USA). First-strand cDNA was synthesized according to the manufacturer’s instructions (Invitrogen, CA, USA). The primer sets for *DIRAS1* were designed to span intronic sequences between adjacent exons to control for genomic DNA contamination. Semi-quantitative reverse transcription PCR (RT-PCR) was amplified for 34 cycles. Glyceraldehyde-3-phosphatedehydrogenas (GAPDH) was used as an internal control. Primer sequences are shown in Additional file 1: Table S1.

### Bisulfite modification, methylation-specific PCR, and bisulfite sequencing

DNA was prepared by the proteinase K method. In brief, cultured cells and fresh tissue samples were digested by DNA digestion buffer (pH 8.0, 10 mM Tris. Cl, 25 mM EDTA, 1% SDS, 100 **μ**g/ml proteinase K) and extracted by phenol/chloroform. Bisulfite treatment was performed as previously described [14]. Methylation-specific PCR (MSP) primers were designed according to genomic sequences around transcriptional start sites (TSS) and synthesized to detect unmethylated (U) and methylated (M) alleles. Bisulfite sequencing (BSSQ) was performed as previously described [15]. BSSQ products were amplified by primers flanking the targeted regions including MSP products. Sequences of the MSP primers and bisulfite sequencing primers are shown in Additional file 1: Table S1. To obtain more evidence supporting our discovery, the expression and the methylation status of *DIRAS1* in The Cancer Genome Atlas database were analyzed in primary colorectal cancer and adjacent tissue samples (Additional file 2: Figure S1).

### Immunohistochemistry

Immunohistochemistry (IHC) was performed in primary colorectal cancer samples and paired adjacent tissue samples. The DIRAS1 antibody was diluted 1:100 (Bioworld Technology, Beijing, China). The staining intensity and extent of the staining area were scored using the German semi-quantitative scoring system as previously described [15].

### Plasmid construction and transfection

Human full-length *DIRAS1* coding sequences (CDS) was amplified and subcloned as described previously [4]. The primers used were 5′-CGCGGATCCATGCCGGAACAGAGTAACG-3′ (F) and 5′-CCGCTCGAGTCACATGAGGGTGCATTTGC-3′ (R). *DIRAS1* expressing lentiviral or empty vectors were packaged using the ViraPower™ lentiviral expression system (Invitrogen, San Diego, CA, USA). Lentivirus was added to the growing medium of DLD1 and RKO cells, and *DIRAS1* stably expressed cells (DLD1-*DIRAS1* and RKO-*DIRAS1* cells) and control cells (DLD1-Vector and RKO-Vector cells) were selected by blasticidin (Invitrogen, San Diego, CA, USA) at a concentration of 5 μg/ml. The selected siRNAs targeting *DIRAS1* sequences were as follows: sense: 5′-CCACCAGGCAAUAACCACATT-3′; antisense: 5′-UCGAUGUUGAGGCUCAUGUTT-3′; negative control sequences were as follows: sense: 5′-UUCUCCGAACGUGUCACGUTT-3′; antisense: 5′-ACGUGACACGUUCGGAGAATT-3′.

### Western blot

Protein preparation and Western blot were performed as described previously [16]. The antibodies for Western blot analysis were as follows: rabbit anti-DIRAS1 (Bioworld Technology, Beijing, China), rabbit anti-MMP2 (Bioworld Technology, Beijing, China), rabbit anti-MMP9 (Bioworld Technology, Beijing, China), and rabbit anti-cleaved caspase 3 (Bioworld Technology, Beijing, China). Rabbit anti-GAPDH (Bioworld Technology, Beijing, China) was used as a control.

### Cell viability detection

Cells were plated into 96-well plates at 1.5 × 10^3^ cells/well, and the cell viability was measured by the MTT assay (KeyGEN Biotech, Nanjing, China) at 0, 24, 48, and 72 h. Absorbance was measured on a microplate reader (Thermo Multiskan MK3, MA, USA) at a wavelength of 490 nm.

### Colony formation assay

Cells were seeded at 500 cells per well in 6-well culture plates in triplicate. The complete growth medium conditioned with blasticidin at 2 μg/ml was exchanged every 48 h. After 2 weeks, cells were fixed with 75% ethanol for 30 min and stained with 0.2% crystal violet (Beyotime, Nanjing, China) for visualization and counting.

### Flow cytometry

*DIRAS1* unexpressed and re-expressed DLD1 and RKO cells were treated with staurosporine (STS) at 100 and 120 ng/ml, respectively, for 24 h [13]. The cells were prepared using the FITC Annexin V Apoptosis Detection Kit I (BD Biosciences, Franklin Lakes, NJ, USA) following the manufacturer’s instructions and then sorted by FACS Calibur (BD Biosciences, Franklin Lakes, NJ, USA).

### Transwell assay

Cells were suspended in serum-free medium. Cells (2 × 10^5^) were seeded into the upper chamber of an 8-μm pore size transwell apparatus (Corning, NY, USA) and incubated for 20 h. Cells that migrated to the lower surface of the membrane were stained with crystal violet and counted in three independent fields. For invasion analysis, cells (2 × 10^5^) were placed into the upper chamber of a transwell apparatus coated with extracellular matrix gel (ECM gel, BD Biosciences, San Jose, CA) and incubated for 48 h. Cells that invaded into the lower membrane surface were stained with crystal violet and counted in three independent fields.

### Generation of colorectal cancer xenografts and assessment of tumor growth

DLD1-*DIRAS1* and control cells (1 × 10^7^cells in 200 μl PBS) were injected subcutaneously into the dorsal right flank of male athymic nude mice. Each group includes eight mice. Subcutaneous tumor volumes (*V*) were measured weekly with digital calipers and calculated using the formula *V* = 1/2 × length × (width)^2^. Mice were sacrificed on the 21st day; tumor weight was measured.

### Statistical analysis

SPSS 18.0 software (IBM, NY, USA) was used for data analysis. All data were presented as means ± standard deviation (SD) and analyzed using the Student’s *t* test. The Chi-squared test and the Fisher’s exact test were used to analyze the association of *DIRAS1* methylation and clinic-pathologic factors, as well as the association of *DIRAS1* expression and the promoter region methylation. The association of *DIRAS1* methylation and overall survival rate of patients were calculated by the Kaplan-Meier method, and differences in survival curve were evaluated using the log-rank test. The correlation between mRNA expression and methylation was performed with the Spearman’s rank correlation coefficient (rho). The value of *P <* 0.05 was considered to be a significant difference.

## Results

### *DIRAS1* is silenced by promoter region hypermethylation in colorectal cancer cells

**Fig. 1:**
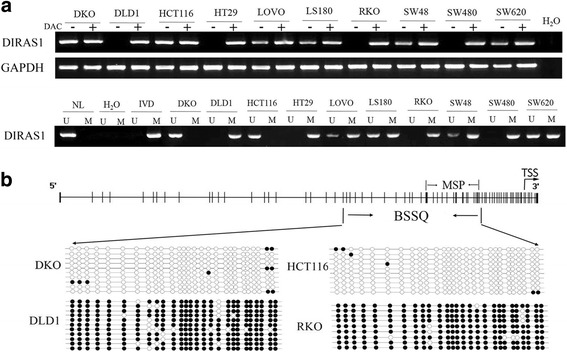
The expression and methylation status of *DIRAS1* in colorectal cancer cells. **a** *Top panel*: semi-quantitative RT-PCR shows *DIRAS1* expression levels in colorectal cancer cell lines: DKO, DLD1, HCT116, HT29, LoVo, LS180, RKO, SW48, SW480, and SW620. *DAC* 5-aza-2′-deoxycytidine, *GAPDH* internal control of RT-PCR, *H*_*2*_ *O* double distilled water. (-) absence of DAC. (+) presence of DAC. *Bottom panel*: MSP results of *DIRAS1* in colorectal cancer cell lines. *U* unmethylated alleles, *M* methylated alleles, *IVD* in vitro methylated DNA, serves as methylation control, *NL* normal lymphocytes DNA, serves as unmethylation control. **b** BSSQ results of *DIRAS1*. MSP PCR product spanned 143 bp in *DIRAS1* promoter region. Bisulfite sequencing region is located 284 bp upstream of transcription start site in the genome. *TSS* transcription start site

To explore the regulation of *DIRAS1* in colorectal cancer, the expression of *DIRAS1* was detected by semi-quantitative RT-PCR in 10 colorectal cancer cell lines. *DIRAS1* was expressed in DKO and HCT116 cells. Reduced expression was found in LoVo, SW48, LS180, and SW620 cells. No expression of *DIRAS1* was detected in DLD1, HT29, RKO, and SW480 cells (Fig. 1a, top panel). Methylation was evaluated by methylation-specific PCR. Complete methylation was found in the promoter region of DLD1, HT29, RKO, and SW480 cells. Partial methylation was detected in LoVo, LS180, SW48, and SW620 cells, and unmethylation was found in DKO and HCT116 cells (Fig. 1a, bottom panel). Methylation results were validated by bisulfite sequencing in DKO, HCT116, DLD1, and RKO cells (Fig. 1b). Promoter region methylation correlated with loss of/reduced expression of *DIRAS1*. Re-expression of *DIRAS1* was induced by DAC, a demethylation agent, in DLD1, HT29, RKO, and SW480 cells (Fig. 1a, top panel). These results suggest that the expression of *DIRAS1* is regulated by promoter region methylation.

### *DIRAS1* is frequently methylated in human primary colorectal cancer, and methylation of *DIRAS1* is associated with poor prognosis

**Fig. 2:**
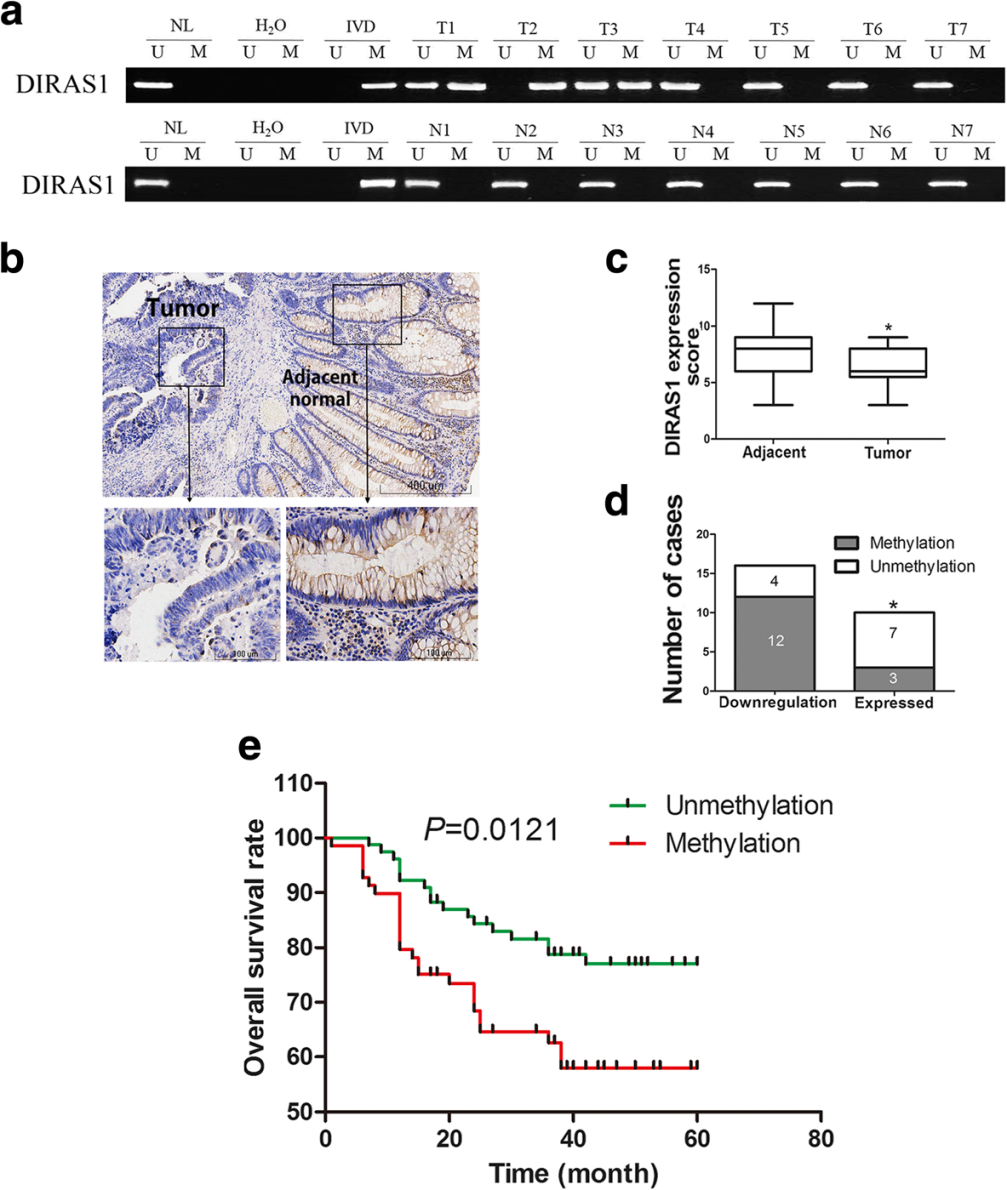
Methylation status and expression of *DIRAS1* in primary colorectal cancer samples. **a** Representative results of MSP for *DIRAS1* in primary colorectal cancer samples and matched adjacent tissue samples. *T* primary colorectal cancer samples, *N* normal colorectal mucosa. **b** Representative IHC results show the expression levels of *DIRAS1* in colorectal cancer and matched adjacent tissue samples (*upper*: ×100; *lower*: ×400). **c** *DIRAS1* expression scores are shown as *box plots*, *horizontal lines* represent the median score; the *bottom* and *top* of the boxes represent the 25th and 75th percentiles, respectively; *vertical bars* represent the range of data. The expression levels of *DIRAS1* were significantly different between adjacent tissue and colorectal cancer samples. (*P < 0.05). **d** The *bar diagram* shows the expression and DNA methylation status of *DIRAS1* in different cancer samples. Reduced expression of *DIRAS1* was significantly associated with promoter region methylation. (*P < 0.05). **e** Kaplan-Meier curves show the association of overall survival rate of colorectal cancer patients with the methylation status of *DIRAS1. Green*, *DIRAS1* unmethylated colorectal cancer patients (*n* = 77, median survival, 25 months); *red*, *DIRAS1* methylated colorectal cancer patients (*n* = 69, median survival, 51 months, *P* = 0.0121, log-rank test)

Methylation of *DIRAS1* was detected in 47.3% (69/146) of primary colorectal cancers, and no methylation was found in 50 cases of non-cancerous colonic tissue samples (Fig. 2a). The expression of *DIRAS1* was evaluated by IHC in 26 matched cancer and adjacent tissue samples. Low-level expression of *DIRAS1* was found in 16 of 26 (61.5%) cancer samples compared to adjacent non-cancerous tissue samples (Fig. 2b, c). In the 16 cancer samples with low level expression of *DIRAS1*, 12 cases were methylated. By contrast in the 10 cancer samples that had normal *DIRAS1* expression levels, 3 cases were methylated. Reduced expression of *DIRAS1* was significantly associated with promoter region methylation (*P <* 0.05, Fig. 2d). These results indicate that *DIRAS1* expression is regulated by promoter methylation in human primary colorectal cancer. Methylation of *DIRAS1* was significantly associated with TNM stage (*P <* 0.05, Table 1) and reduced 5-year overall survival (*P =* 0.0121, Fig. 2e). These results demonstrate that methylation of *DIRAS1* may serve as a marker of poor prognosis in human colorectal cancer.

**Table 1:** Association of DIRAS1 methylation with clinicopathologic features in colorectal tumor

Clinical factor	DIRAS1 methylation status		*P* value
	Unmethylated	Methylated	
Gender			0.72
Male	53	45	
Female	24	24	
Age (year)			0.51
>60	40	40	
≤60	37	29	
Tumor size (cm)			0.86
>5	51	44	
≤5	26	25	
Differentiation			
Well	1	1	0.88
Moderate	60	44	
Poor	16	24	
Tumor invasion			0.12
T_1_	0	0	
T_2_	16	6	
T_3_	58	59	
T_4_	3	4	
Lymph node metastasis			0.07
N_0_	48	32	
N_1_	29	37	
TNM stage			0.02*
I-II	47	28	
III-IV	30	41	

Chi-square test; *P < 0.05

By searching the Cancer Genome Atlas (TCGA) database (https://cancergenome.nih.gov/), 217 cases of primary colorectal cancer and 16 cases of adjacent tissue samples were found to have both *DIRAS1* gene expression and methylation data, which were obtained from RNA sequencing and HMK450 methylation array, respectively. The promoter region methylation was inversely associated with DIRAS1 expression in colorectal cancer (*R* = −0.278, *P <* 0.0001, Additional file 2: Figure S1B). The levels of DNA methylation were significantly higher in colorectal cancer samples compared to adjacent tissue samples (*P* < 0.0001, Additional file 2: Figure S1C). No association was found between 5-year overall survival and DIRAS1 expression (*P >* 0.05, Additional file 2: Figure S1D or methylation (*P >* 0.05, Additional file 2: Figure S1E). The reason is that TCGA data was collected from different institutes and their therapeutic regimen maybe different.

### *DIRAS1* inhibits cell proliferation, migration, and invasion in colorectal cancer

**Fig. 3:**
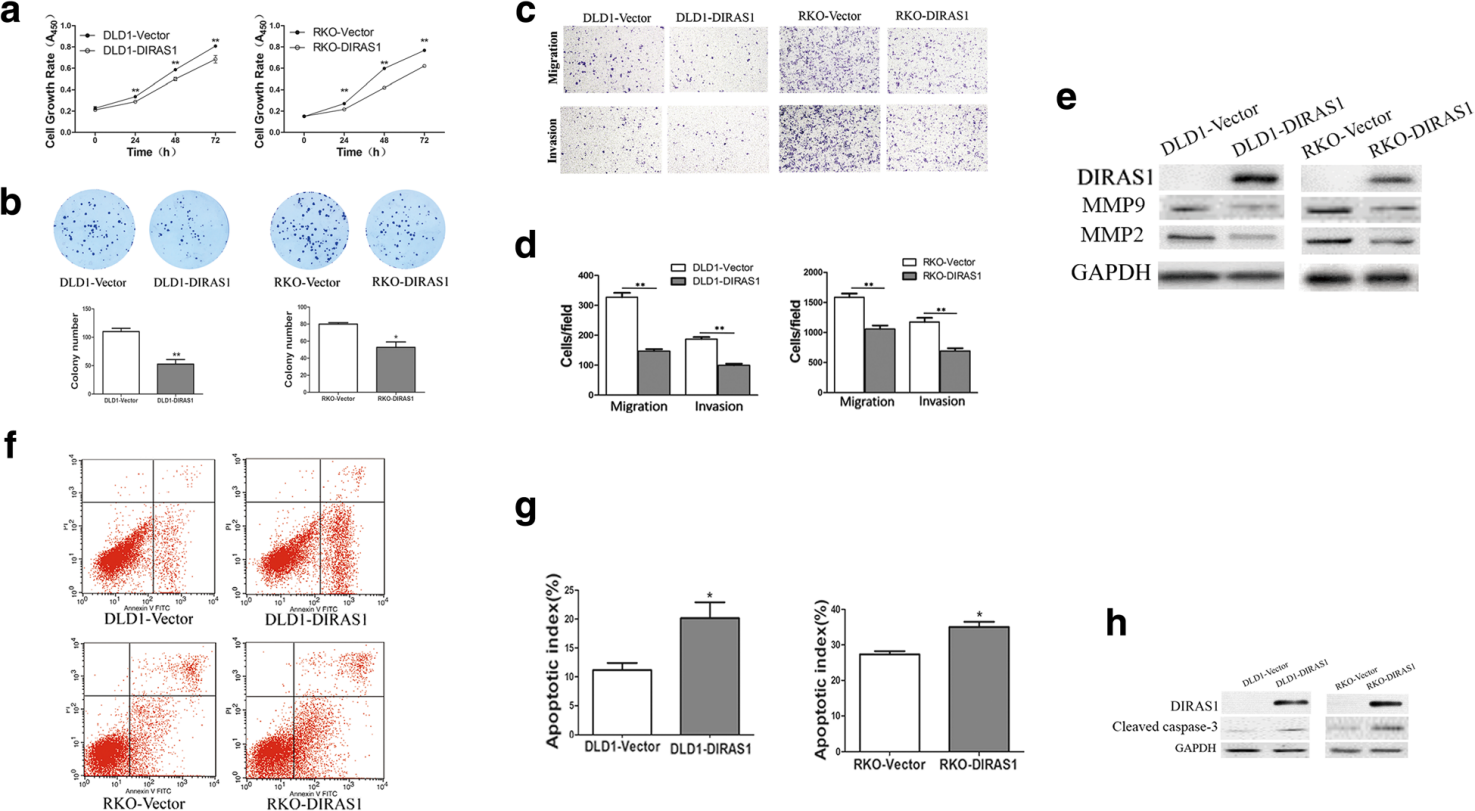
The effects of *DIRAS1* on colorectal cancer cell proliferation, migration, invasion, and apoptosis in *DIRAS1* re-expressed colorectal cancer cells. **a** *Growth curves* represent cell viability analyzed by the MTT assay in *DIRAS1* re-expressed and unexpressed DLD1 and RKO cells. The experiments were performed in triplicate. (**P < 0.01). **b** Colony formation assays show colony numbers in DLD1 and RKO cells before and after re-expression of *DIRAS1*. Each experiment was repeated three times. The average number of tumor clones is represented by bar diagram. (* *P <* 0.05, ** *P <* 0.01). **c** Cell migration and invasion results in DLD1 and RKO cells before and after re-expression of *DIRAS1*, **d** ratios are presented by *bar diagram*. Each experiment was repeated three times. (**P < 0.01). **e** The expression levels of *DIRAS1*, MMP2, and MMP9 were detected by Western blot in *DIRAS1* unexpressed and re-expressed DLD1 and RKO cells. **f**, **g** Representative images of Annexin-V and PI double staining in *DIRAS1* unexpressed and re-expressed colorectal cancer cells (*P < 0.05). **h** Western blots show the levels of cleaved caspase-3 in *DIRAS1* re-expressed and unexpressed colorectal cancer cells. The levels of cleaved caspase-3 were increased in *DIRAS1* re-expressed DLD1 and RKO cells

**Fig. 4:**
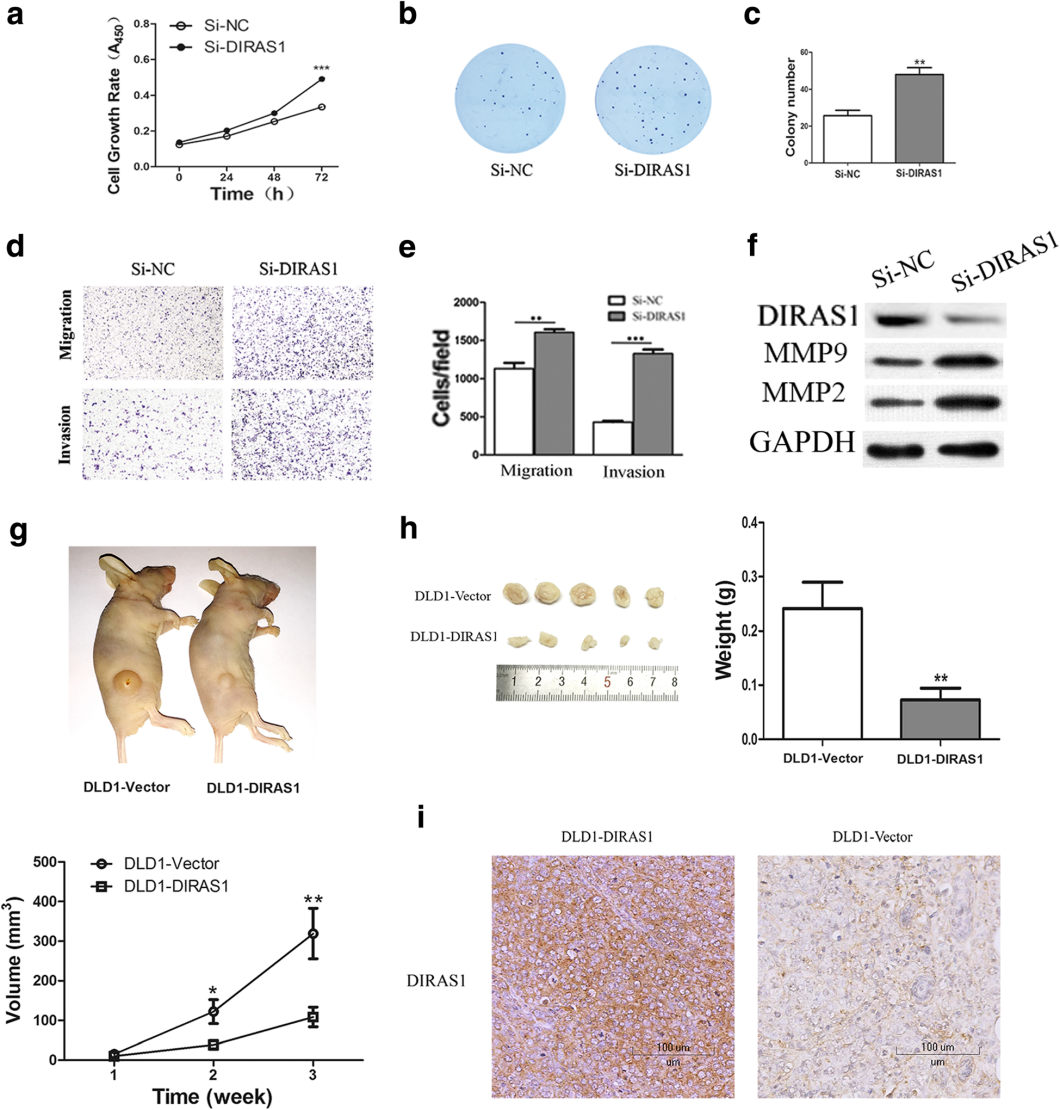
The function of *DIRAS1* in *DIRAS1* highly expressed HCT116 cells and in vivo study of *DIRAS1* in colorectal cancer cell xenograft mice. **a** *Growth curves* represent cell viability analyzed and by the MTT assay in *DIRAS1* highly expressed HCT116 cells before and after knockdown of *DIRAS1*. The experiments were performed in triplicate. (***P < 0.001). **b** Colony formation assays show colony numbers in *DIRAS1* highly expressed HCT116 cells before and after knockdown of *DIRAS1*. Each experiment was repeated three times, **c** the average number of tumor clones is represented by bar diagram. (**P < 0.01). **d** Cell migration and invasion results in *DIRAS1* highly expressed HCT116 cells before and after knockdown of *DIRAS1*, **e** ratios are presented by *bar diagram*. Each experiment was repeated three times. (**P < 0.01, ***P < 0.001). **f** The expression levels of *DIRAS1*, MMP2, and MMP9 were detected by Western blot in *DIRAS1* highly expressed HCT116 cells before and after knockdown of *DIRAS1*. Knockdown of *DIRAS1* by siRNA was performed to validate the results in *DIRAS1* highly expressing HCT116 cells. *siNC DIRAS1* highly expressed HCT116 cells, *siDIRAS1* knockdown of *DIRAS1* in HCT116 cells. **g** Volumes of xenograft tumors in *DIRAS1* re-expressed and unexpressed in DLD1 cells after 2 and 3 weeks, respectively (*P < 0.05, **P < 0.01). **h** Weights of xenograft tumors in *DIRAS1* re-expressed and unexpressed DLD1 and RKO cells after incubation for 3 weeks (**P < 0.01). **i** IHC shows the expression of *DIRAS1* in *DIRAS1* re-expressed and unexpressed DLD1 cell xenografts (×400)

To evaluate the effects of *DIRAS1* on cell proliferation, cell viability was detected by MTT and colony formation assays. The OD values at 72 h were 0.806 ± 0.011 vs 0.6843 ± 0.035 in DLD1 cells (*P <* 0.01) and 0.768 ± 0.004 vs. 0.621 ± 0.003 (*P <* 0.01) in RKO cells before and after restoration of *DIRAS1* expression (Fig. 3a). The results demonstrated that *DIRAS1* inhibits cell viability in colorectal cancer cells. The clone numbers were 110.0 ± 5.8 vs. 52.7 ± 8.2 in DLD1 cells (*P <* 0.01) and 80.0 ± 1.7 vs. 52.7 ± 6.4 in RKO cells (*P <* 0.05) before and after restoration of *DIRAS1* expression (Fig. 3b). The results suggest that *DIRAS1* suppresses colorectal cancer cell growth. To further validate above results, siRNA technique was employed in *DIRAS1* highly expressed HCT116 cells. The OD values were 0.335 ± 0.005 vs. 0.4920 ± 0.012 in HCT116 cells before and after knockdown of *DIRAS1* (*P <* 0.001) (Fig. 4a). The results suggest that *DIRAS1* inhibits cell viability in CRC cells. The clone numbers were 25.7 ± 3.0 vs. 48.0 ± 3.6 in HCT116 cells before and after knockdown of *DIRAS1* in HCT116 cells (*P <* 0.01, Fig. 4 b, c). It suggests that *DIRAS1* suppresses colorectal cancer cell growth.

To explore the effect of *DIRAS1* on cell migration, the transwell assay in the absence of ECM gel coating was employed. The numbers of migrated cells for each high-power field under the microscope were 327.0 ± 14.8 vs. 147.0 ± 6.4 in DLD1 cells (*P <* 0.01) and 1586.0 ± 62.5 vs. 1058.0 ± 58.61 in RKO cells (*P <* 0.01) before and after restoration of *DIRAS1* expression (Fig. 3c top panel, Fig. 3d). The results demonstrate that *DIRAS1* inhibits colorectal cancer cell migration. Next, the transwell assay with ECM coating was employed to evaluate the effect of *DIRAS1* on cell invasion. The numbers of invasive cells for each high-power field under the microscope were 187.3 ± 6.5 vs. 99.7 ± 5.2 in DLD1 cells (*P <* 0.01) and 1173.0 ± 71.2 vs. 691.7 ± 45.3 in RKO cells (*P <* 0.01) before and after restoration of *DIRAS1* expression (Fig. 3c bottom panel, Fig. 3d).

In *DIRAS1* highly expressed HCT116 cells, siRNA technique was employed for transwell study. The numbers of migrated cells for each high-power field under the microscope were 1134.0 ± 75.16 vs. 1605.0 ± 40.37 (*P <* 0.01) cells before and after knockdown of *DIRAS1* in HCT116 cells (Fig. 4d top panel, Fig. 4e). It further suggests that *DIRAS1* inhibits cell migration in CRC. The numbers of invasive cells for each high-power field under the microscope were 428.7 ± 19.6 vs. 1329 ± 54.0 before and after knockdown of *DIRAS1* in HCT116 cells (*P <* 0.001) (Fig. 4d bottom panel, Fig. 4e). The result suggests that *DIRAS1* inhibits cell invasion in colorectal cancer.

The expression levels of MMP2 and MMP9 were inhibited by re-expression of *DIRAS1* in DLD1 and RKO cells (Fig. 3e), while they were increased after knockdown of *DIRAS1* in *DIRAS1* expressing HCT116 cells (Fig. 4f). These results suggest that *DIRAS1* impedes colorectal cancer cell invasion.

### *DIRAS1*-induced apoptosis in human colorectal cancer cells

The effects of *DIRAS1* on cell cycle and apoptosis were analyzed by flow cytometry in human colorectal cancer cells. No significant differences in cell phase distribution were found in DLD1 and RKO cells before and after re-expression of *DIRAS1* (all *P* > 0.05, data not shown). The ratios of apoptosis were 11.16 ± 1.25% vs. 20.15 ± 2.75% in DLD1 cells (*P <* 0.05) and 28.92 ± 2.78% vs. 38.12 ± 0.87% in RKO cells (*P <* 0.05) before and after re-expression of *DIRAS1* under the treatment of STS (Fig. 3 f, g). As shown in Fig. 3h, the levels of cleaved caspase-3 were increased after re-expression of *DIRAS1* in DLD1 and RKO cells.

### *DIRAS1* suppresses tumor growth in xenograft mice

To further validate the role of *DIRAS1* in colorectal cancer, *DIRAS1* unexpressed and re-expressed DLD1 cells were used to establish xenograft tumors in mice. The tumor volumes were 319.5 ± 64.0 mm^3^ in *DIRAS1* unexpressed DLD1 cell xenograft mice and 108.6 ± 24.78 mm^3^ in *DIRAS1* re-expressed DLD1 cell xenograft mice. The tumor volumes were smaller in *DIRAS1* re-expressed DLD1 cell xenograft mice compared to *DIRAS1* unexpressed DLD1 cell xenograft mice (*P <* 0.01, Fig. 4g). The tumor weights were 241.0 ± 48.9 mg in *DIRAS1* unexpressed DLD1 cell xenograft mice and 72.7 ± 21.6 mg in *DIRAS1* re-expressed DLD1 cell xenograft mice (*P <* 0.01, Fig. 4h). The tumor weights were lower in *DIRAS1* expressed DLD1 cell xenograft mice compared to *DIRAS1* unexpressed DLD1 cell xenograft mice. These results suggest that *DIRAS1* suppressed colorectal cancer cell growth in vivo.

## Discussion

The Ras superfamily is divided into five subfamilies according to their sequence homology and biochemical properties. The five families are Ras, Rab, Rho, Ran, and Arf. Ras members may activate downstream signaling by binding to GTP or serve as an inactive form by binding to GDP. When bound to GTP, Ras proteins associate with effectors, resulting in the propagation of downstream signaling. Most members of this superfamily have been widely studied and identified as oncoproteins [17, 18]. *DIRAS1* may serve as a competitive inhibitor of Ras and antagonize Ras-mediated ERK1/2 signaling to promote cell apoptosis and suppress cell invasion. The expression levels of *DIRAS1* were found to be reduced in human breast cancer and esophageal cancer [13, 19]. In this study, we found that *DIRAS1* is frequently methylated in human CRC, and the expression of *DIRAS1* is regulated by promoter region methylation. In TCGA database, the promoter region methylation was inversely associated with *DIRAS1* expression in colorectal cancer. The levels of DNA methylation were significantly higher in colorectal cancer samples compared to adjacent tissue samples according to HMK450 methylation array data in human colorectal cancer. These data further supported our results. Methylation of *DIRAS1* was associated with TNM stage and short survival. These results suggest that methylation of *DIRAS1* may serve as a marker of poor prognosis in human colorectal cancer. In our studied cohort, patients were received different chemotherapeutic regimens. It limits to further analyze the association of *DIRAS1* methylation and each chemical agent or therapy. The consequence of each regimen may be different in CRC treatment, and TCGA data was collected from different institutes by using different regimens. It may affect to evaluating the association between the 5-year overall survival and *DIRAS1* expression or methylation. A well-designed large cohort study is necessary to further validate that *DIRAS1* methylation is a marker of poor prognosis in CRC by using a unique detection method.

*DIRAS1* inhibits cell proliferation, migration, and invasion and induces apoptosis in colorectal cancer cells. *DIRAS1* suppresses colorectal cancer cell xenograft growth in vivo. Collectively, our results suggest that *DIRAS1* is a tumor suppressor in human CRC.

## Conclusions

*DIRAS1* is frequently methylated in human colorectal cancer, and the expression of *DIRAS1* is regulated by promoter region methylation. Methylation of *DIRAS1* is a marker of poor prognosis in human colorectal cancer. Methylation of *DIRAS1* may promote colorectal carcinogenesis and progression.

## Additional files

Additional file 1: Table S1. Nucleotide sequences of the primers used in this study. (XLS 25 kb)

Additional file 2: Figure S1. Methylation status and expression of DIRAS1 in primary colorectal cancer and adjacent samples from TCGA. (A) The correlation of methylation of each CpG site (HM450) and expression of *DIRAS1*. (B) The methylation status of the CpG site (cg05228284, HM450) is correlated to loss of/reduced *DIRAS1* expression in 217 cases of colorectal cancer samples and 16 cases of adjacent samples. (*R* = −0.278, *P* < 0.0001). (C) The methylation status of the CpG site (cg05228284, HM450) is correlated to loss of/reduced *DIRAS1* expression in 234 cases of colorectal cancer. (*P* < 0.0001). (D) Kaplan-Meier curves show the association of overall survival rate of colorectal cancer patients with the methylation status of CpG site (cg05228284, HM450). (*P* > 0.05). (E) Kaplan-Meier curves show the association of overall survival rate of colorectal cancer patients with the expression levels of *DIRAS1*. (*P* > 0.05). (TIF 572 kb)

## Abbreviations

BSSQ: Bisulfite sequencing
CRC: Colorectal cancer
DAC: 5-Aza-2′-deoxycytidine
DKO: DNMT1 and DNMT3b double knockout
ECM: Extracellular matrix
GAPDH: Glyceraldehyde-3-phosphatedehydrogenas
IHC: Immunohistochemistry
M: Methylated
MSP: Methylation-specific PCR
Rig: Ras-related inhibitor of cell growth
SD: Standard deviation
STS: Staurosporine
TSS: Transcriptional start sites
U: Unmethylated
