# Supplementary figures and images for: Methylation of DIRAS1 promotes colorectal cancer progression and may serve as a marker for poor prognosis
Source: Clin Epigenetics. 2017 May 10;9:50. doi: 10.1186/s13148-017-0348-0 (PMC5424295; doi:10.1186/s13148-017-0348-0)

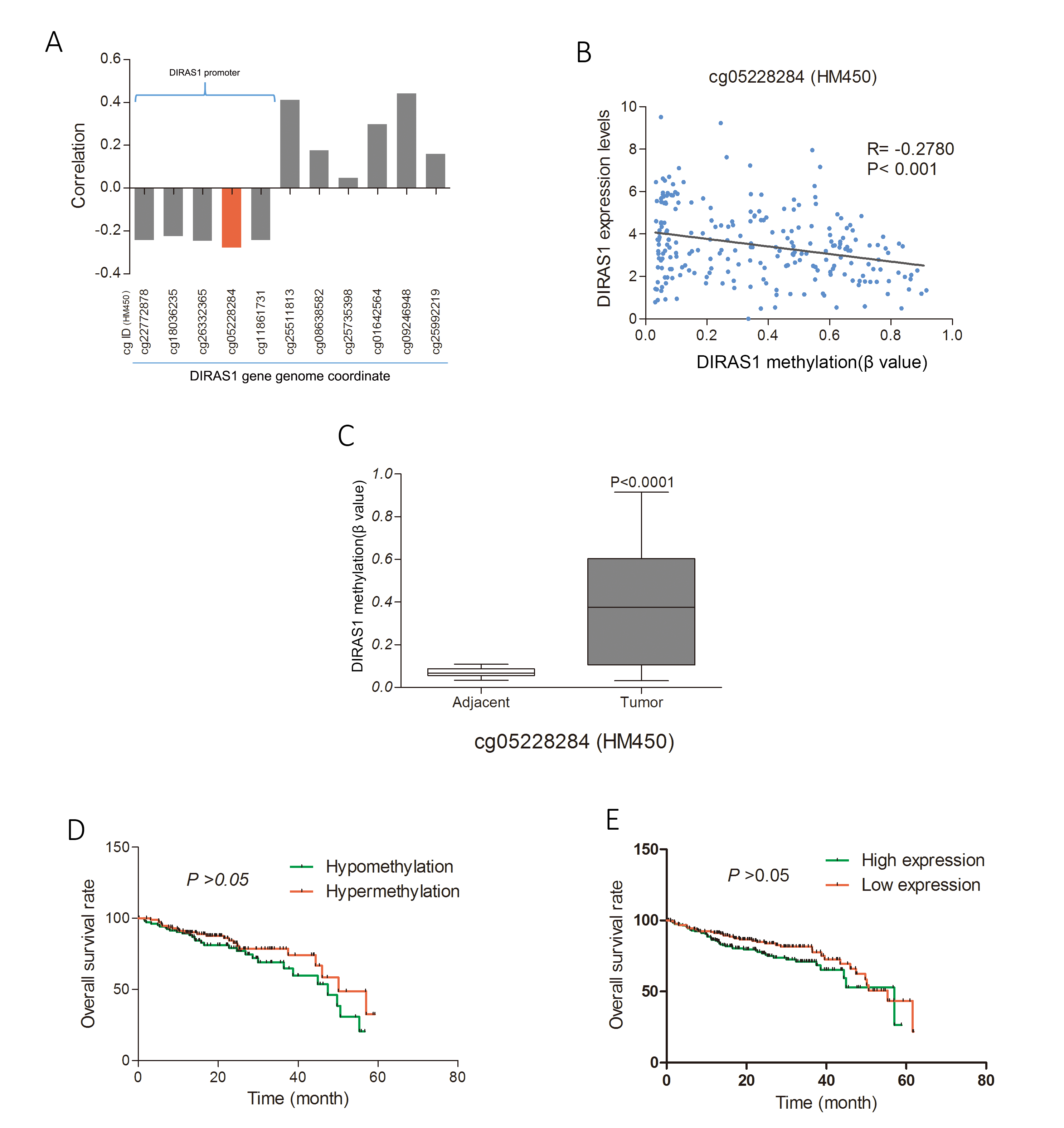

Supplement: Supplementary file 2 — Methylation status and expression of DIRAS1 in primary colorectal cancer and adjacent samples from TCGA. (A) The correlation of methylation of each CpG site (HM450) and expression of DIRAS1. (B) The methylation status of the CpG site (cg05228284, HM450) is correlated to loss of/reduced DIRAS1 expression in 217 cases of colorectal cancer samples and 16 cases of adjacent samples. (R = −0.278, P < 0.0001). (C) The methylation status of the CpG site (cg05228284, HM450) is correlated to loss of/reduced DIRAS1 expression in 234 cases of colorectal cancer. (P < 0.0001). (D) Kaplan-Meier curves show the association of overall survival rate of colorectal cancer patients with the methylation status of CpG site (cg05228284, HM450). (P > 0.05). (E) Kaplan-Meier curves show the association of overall survival rate of colorectal cancer patients with the expression levels of DIRAS1. (P > 0.05). (TIF 572 kb) [file 13148_2017_348_MOESM2_ESM.tif]
